# Circulating plasma serine^208^-phosphorylated troponin T levels are indicator of cardiac dysfunction

**DOI:** 10.1111/jcmm.12112

**Published:** 2013-08-02

**Authors:** Emilie Dubois-Deruy, Aude Belliard, Paul Mulder, Maggy Chwastyniak, Olivia Beseme, Jean-Paul Henry, Christian Thuillez, Philippe Amouyel, Vincent Richard, Florence Pinet

**Affiliations:** a INSERM, U744Lille, France; b Institut Pasteur de LilleLille, France; c University of Lille 2, IFR141Lille, France; d INSERM, U1096, Institute for Biomedical Research, IFRMP 23, Rouen University HospitalRouen, France; e Centre Hospitalier Regional et Universitaire Cardiologic hospitalLille, France

**Keywords:** Rat, myocardial infarction, plasma, biomarker, phosphorylated troponin T, ivabradine, heart failure

## Abstract

Heart failure (HF) following myocardial infarction (MI) is characterized by progressive alterations of left ventricular (LV) structure and function, named LV remodelling. Although several risk factors such as infarct size have been identified, HF remains difficult to predict in clinical practice. Recently, using phosphoproteomic technology, we found that serine^208^-phosphorylated troponin T (P-Ser^208^-TnT) decreases in LV of HF rats. Our aim was to determine the performance of P-Ser^208^-TnT as plasma biomarker of HF compared to conventional cardiac biomarkers such as B-type natriuretic peptide (BNP), cardiac troponin I (cTnI), C-reactive protein (CRP) or tissue inhibitor of metalloproteinase I (TIMP-1) measured by x-MAP technology, as well as its capacity to reflect a pharmacological improvement of HF. We observed a significant increase of BNP, TnT and cTnI levels and a significant decrease of P-Ser^208^-TnT and TIMP-1 in the plasma of 2-month-MI rats compared with control rats with no modulation of CRP level. Circulating levels of P-Ser^208^-TnT were shown to be associated with most of the echocardiographic and haemodynamic parameters of cardiac function. We verified that the decrease of P-Ser^208^-TnT was not because of an excess of phosphatase activity in plasma of HF rats. Two-month-MI rats treated with the heart rate reducing agent ivabradine had improved LV function and increased plasma levels of P-Ser^208^-TnT. Thus, circulating phosphorylated troponin T is a highly sensitive biological indicator of cardiac dysfunction and has the potentiality of a new biomarker of HF post-MI, and of a surrogate marker for the efficacy of a successful treatment of HF.

## Introduction

Left ventricular (LV) remodelling following myocardial infarction (MI) is characterized by progressive alterations of LV structure and function, named LV remodelling [1]. Despite significant improvements in MI management, heart failure (HF) remains a frequent complication of LV remodelling [2]. Although several mechanisms implicated in LV remodelling and aggravation of LV dysfunction have been identified, such as infarct size, hypertrophy, fibrosis, apoptosis and proteolytic activation, as well changes in contractile proteins and/ or proteins involved in calcium handling [3–5], the exact molecular determinants of LV dilation and of its functional consequences remain imperfectly known. Although several risk factors have been identified, HF remains difficult to predict in clinical practice. The only biomarker clinically used in MI patients is B-type natriuretic peptide (BNP) whose concentration helps to predict the risk of HF [6], but data on its ability to predict LV remodelling are discordant [7–9]. Recently, the REVE-2 (REmodelage VEntriculaire-2) study was designed to test if plasma levels of three cardiac biomarkers, BNP, troponin I (TnI) and C-reactive protein (CRP) might be correlated with post-MI LV remodelling (defined as >20% increase in end diastolic volume with 1-year echocardiographic follow-up). At baseline, significant univariate relations were found between LV remodelling and the three biomarkers, however, in multivariate analysis, none of the three biomarkers were independently predictive of LV remodelling [10]. During the follow-up, high BNP levels and persistently detectable levels of TnI were associated with LV remodelling, suggesting that determination of BNP and cTnI during the follow-up can help refine risk stratification for LV remodelling [10].

For a better understanding of the molecular mechanisms involved in post-MI LV remodelling, experimental animal models can be used offering easy access to heart tissue and plasma from the same animal and less heterogeneity and variability than humans. Induction of anterior MI in rat has been shown to lead to LV remodelling and dysfunction and subsequently to HF [11]. This model has been characterized to be an accurate representation of LV remodelling that occurs in humans. Major advances in the prevention of LV remodelling, such as angiotensin-converting enzyme inhibitors were initially discovered in this experimental model [12]. Using this experimental model of MI and phosphoproteomic approach, we recently discovered that LV remodelling is associated with decreased levels of myocardial and plasma serine^208^-phosphorylated troponin T (P-Ser^208^-TnT) and confirmed the same decrease in patients with high LV remodelling, suggesting that the level of circulating phosphorylated TnT could be new biomarker of LV remodelling and may help to predict the development of HF after MI [13].

To ensure the potentiality of P-Ser^208^-TnT as biomarker, we need to verify that the decrease of P-Ser^208^-TnT in plasma of 2-month-MI rats was not because of an increase of phosphatase activity. We also need to compare its performance with other cardiac markers in the experimental animal model of HF after MI. Up to now, there is no information on plasma levels of BNP, TnI and CRP, three proteins that have been recently measured in patients with post-MI LV remodelling [10]. We used the multiplex technology to quantify BNP, TnI, CRP, TIMP-1. This new approach allows using less sample than a classical ELISA with the same sensitivity, but a higher dynamic range and without any cross-reactivity between them. We also selected TIMP-1 as its level was associated to cardiovascular risk factors and cardiac echocardiographic alterations [14].

The purpose of this study was then to assess (*i* ) the appropriateness of plasma levels of TnT and P-Ser^208^-TnT to monitor disease progression in an experimental animal model of HF; (*ii* ) to compare their performance to circulating markers of cardiovascular disorders: BNP, TnI, CRP and TIMP-1; (*iii* ) to determine if the plasma levels of P-Ser^208^-TnT correlate with a pharmacological improvement of cardiac dysfunction and remodelling in this model.

## Materials and methods

### Animals

All experiments performed in this study conformed to the *Guide for the care and use of laboratory animals* published by the US National Institutes of Health (NIH publication No. 85-23, revised in 1996). The use of animals and experimental protocols were performed under supervision of person with habilitation to perform experiment of live animals (F. Pinet: 59-350126, exp. Date: 22 June 2016).

Before surgery, animals were anesthetized (sodium methohexithal, 50 mg/kg IP), while analgesia was administered before (xylazine 5 mg/kg IP) and 1 hr after surgery (xylazine 50 mg/kg subcutaneously). Myocardial infarction was induced in 10-week-old male Wistar rats (*n* = 34) (Charles River, L'Arbresle, France) by left coronary ligation according to the method of Pfeffer *et al*. [12] and modified by Mulder *et al*. [11]. Another 16 rats underwent the same protocol except that the snare was not tied, this was the sham group. All rats were allowed standard rat chow and drinking water *ad libitum* and maintained on a 12-hr/12-hr light/dark cycle. Eight MI-rats received ivabradine given as food additive for 2 months at a dose of 10 mg/kg/day as previously described [15].

Two months after surgery, echocardiography and haemodynamic measurements were performed in anesthetized animals (sodium methohexithal, 50 mg/kg IP). After the haemodynamic assessment, rats were killed by an overdose of sodium pentobarbitall (500 mg/kg IP). Left ventricles (LV) and plasma were collected in glass tubes with EDTA, immediately frozen in liquid nitrogen and kept at −80°C until analysis.

Echocardiographic analysis was performed as described previously [11, 13, 16, 17]. 2D images and 2D-guided M-mode, Doppler M mode, and pulsed-wave Doppler recordings were obtained from parasternal short-axis (level of papillary muscles) views, using a Vivid 7 Ultrasound (GE Healthcare, Rouen, France) echograph with a M12L linear probe operated at 14 MHz, and analysed using Echopac PC software. Left ventricles end-diastolic diameter (EDD) and end-systolic diameter (ESD) were measured by the leading-edge convention, and used to calculate fractional shortening (FS) through the equation FS = [(EDD − ESD)/EDD] × 100. Velocity-time integral (VTI) was measured at the level of the pulmonary artery by pulse-wave Doppler. Stroke volume was calculated as VTI × [π × (left ventricular out-flow diameter/2)^2^], and cardiac output was calculated as stroke volume × heart rate.

The mean of three consecutive cardiac cycles was used for all measurements in each animal. Measurements, performed by a single echocardiographer blinded to the treatment groups, were made in accordance with the conventions of the American Society of Echocardiography.

For haemodynamic measurement, rats were anesthetized with sodium methohexithal (50 mg/kg IP). The right carotid artery was cannulated with micromanometer-tipped catheters (SPR 407, Millar Instruments, Houston, TX, USA) advanced into the aorta for the recording of arterial pressure. The aortic catheter was then advanced into the LV for the recording of LV end-systolic and end-diastolic pressures, LV dP/dt min/max, LV relaxation constant Tau (Weiss method).

### LV protein extraction

Heart LV proteins were extracted from 100 mg of frozen tissue using Dounce-Potter homogenization in ice-cold 40 mmol/l Tris-HCl, pH 9.5 containing anti-proteases (one tablet for 10 ml buffer, Complete™ EDTA-free, Roche Applied Science, Meylan, France) and serine/threonine protein phosphatases inhibitors (1/100, phosphatase inhibitors (1/100, Phosphatase inhibitor cocktail 3, Sigma-Aldrich, L'Isle dabeau, France). Homogenates were then shaken for 1 hr at 4°C prior to centrifugation (14,000 × g for 15 min. at 4°C). Protein concentration was determined in supernatants using Bradford assay and samples were stored in aliquots at −80°C until experiments were performed.

### Western blot analysis

Western blot was performed for TnT, P-Ser^208^-TnT, protein phosphatase 2A (PP2A) and tubulin. The primary antibodies were polyclonal antibodies against the 202–215 sequence of rat TnT, specific either for phosphorylated Ser^208^-TnT (AQTERK(pS)GKRQTER) and non-phosphorylated TnT (AQTERKSGKRQTER) used at a dilution of 1/1000. Each polyclonal antibody was purified with the non-phosphorylated peptide for TnT detection and with the phosphorylated peptide for phosphorylated TnT detection as previously characterized [Fig. S2 in 13]. The other antibodies used were PP2A (mouse species, clone 1D6, 05-421, dilution 1/5000; Millipore, Guyancourt, France) and alpha-tubulin (mouse species, clone B-7, sc-5286, dilution 1/10000; Santa-Cruz, Heidelberg, Germany).

Proteins from LV (50 μg) and from plasma (1 μl) were separated by SDS-PAGE (12% acrylamide) and transferred on 0.4 5 μm Hybond™ nitrocellulose membrane (GE Healthcare). Equal total proteins loads were confirmed by Ponceau red [0.1% Ponceau (Sigma-Aldrich), 5% acetic acid (v/v)] staining of the membranes. We also verified equal LV and plasma proteins for tubulin and PP2A, respectively, in the case of ivabradine experiments. The blots were then washed in TBS-Tween, saturated in 5% BSA (v/w) for TnT blots and in 5% non-fat dry milk (v/w) for tubulin and PP2A. The membranes were incubated overnight in the respective blocking solution with primary antibodies against specific proteins at the dilution described above. The blots were then washed for 10 min. repeated 5 times in TBS-Tween 0.2% and then incubated with a horseradish peroxidase labelled secondary antibody for 1 hr diluted in blocking solution. The secondary antibodies used for western blot analysis were ECl™ anti-rabbit IgG horseradish peroxidase (NA934, dilution 1/10000, GE Healthcare) for TnT and P-Ser^208^-TnT and ECl™ anti-mouse IgG horseradish peroxidase (NA931, dilution 1/5000, GE Healthcare) for PP2A and tubulin. Membranes were then washed for 10 min. repeated 5 times in TBS-Tween 0.2% before incubation with enhanced chemiluminescence (ECl™) western blotting detection reagents (GE Healthcare). The Chemidoc® camera (Biorad, Marnes-la-Coquette, France) was used for blots exposition *and* imaging. Quantity One® 16D analysis software was used for the acquisition and quantification of intensity values from blot images. The same area is selected for each sample analysed in the same membrane and the density is calculated. In each western blot, an internal standard was included and used for the normalization of density from one membrane to another membrane. Normalized (inter and intra membrane) intensity values were used to evaluate differences between TnT and P-Ser^208^-TnT expression levels in sham- compared to 2-month-MI rats and 2-month-MI rats treated or not by ivabradine.

### Multiplexing analysis

B-type natriuretic peptide, TnI, CRP and TIMP-1 were measured in plasma samples using a Multi Analyte Profiling Kit for simultaneous quantitative detection of rat cardiovascular biomarkers, according to the manufacturer's instructions (Rat CVD1 Panel 1, RCVD1-89K and Rat/Mouse CRP Single Plex, MCVD77K1CRP, Millipore).

The filter plate was pre-wet with 200 μl of wash buffer for 10 min. After removing, 25 μl of each standard, quality control and plasma samples and 25 μl of assay buffer were added. Plasma samples required a fourfold dilution for BNP, TnI and TIMP-1 measurements and a 70,000-fold dilution for CRP. Twenty-five microlitre of beads was added and incubated under shaking for 18 hrs at 4°C. Plates were washed 2 times with 200 μl wash buffer and incubated with 25 μl of detection antibody for 2 hrs. Twenty-five microlitre of streptavidin-phycoerythrin was added for 30 min. Plates were then washed 2 times with 200 μl of wash buffer and beads were resuspended with 100 μl of sheat fluid for 5 min. before running. The samples were analysed using the Bio-Plex system (Bio-Rad Laboratories, Hercules, CA, USA) according to the instructions from the manufacturer. Analysis of experimental data was performed using four-parameter logistic curve fitting to the standard analysis curves. The intra- and inter-assay coefficients of variation were <10% for TIMP -1 and <20% for BNP, TnI and CRP.

### Phosphatase activity assay

Protein phosphatase activity in plasma of rats was determined by ELISA according to manufacturer's instructions (ref 22501, Bio-medical assay). The ELISA has a sensitivity of 0.5 U/l. Each sample was analysed in duplicate.

### Statistical analysis

Variables were expressed as mean ± SEM or as median with 25th and 75th percentiles. Differences between sham and 2-month-MI treated or not with ivabradine groups were assessed by Wilcoxon test. The relations between parameters and biomarkers values were tested with spearman correlation. A value of *P* ≤ 0.05 was considered statistically significant. Receiver operating characteristic (ROC) curves were calculated for each biomarker. All the statistical analyses were performed with R software (version 2.12.0, release of 2010-10-15).

## Results and discussion

The aim of this study was to determine the performance of P-Ser^208^-TnT as potential biomarker of LV remodelling post-MI, compared to other cardiac markers. This biomarker was discovered using phosphoproteomic [18] and an experimental rat model of MI [11], and the decrease of P-Ser^208^-TnT was confirmed in patients with high LV remodelling [13]. The interest of experimental model of MI in animals is the easy access to heart tissue and biological fluids in the same animal. For that purpose, a total of 50 rats (16 sham, 34 2-month-MI) were included in the study.

### Cardiac and biological parameters of 2-month-MI rats

Table 1 details echocardiographic, haemodynamic and histomorphometric parameters measured in sham- and 2-month-MI rats. Myocardial infarction induced a significant increase in LV end-diastolic pressure (LVEDP), LV end-systolic diameter (LVESD), LV end-diastolic diameter (LVEDD), E/A, the relaxation constant Tau and dP/dt_min_ and a significant decrease in cardiac output, stroke volume, fractional shortening (FS) and dP/dt_max_. No difference was observed for LV end-systolic pressure (LVESP) and heart rate (Table 1). Myocardial infarction also induced a significant increase in heart weight (HW), right (RVW) and left ventricular weight (LVW), as well as atria (AV) and lung weight (LW), without modification of body weight (BW) (Table 1). All these results are in agreement with a myocardial hypertrophy, associated with HF in rat with left coronary ligation after 2 months.

**Table 1:** Echographic, haemodynamic and histomorphometric parameters in sham- and 2-month-MI rats

Parameters	Sham-rats (*n* = 16)	2-month-MI rats (*n* = 19)
LVESP (mm Hg)	120 ± 6	113 ± 5
LVEDP (mm Hg)	0.73 ± 0.28	6.10 ± 0.58‡
LVESD (mm)	3.17 ± 0.09	8.36 ± 0.22‡
LVEDD (mm)	6.24 ± 0.08	9.89 ± 0.23‡
E/A	1.23 ± 0.12	2.19 ± 0.21‡
Tau (ms)	3.38 ± 0.11	7.36 ± 0.90‡
Cardiac output (ml/min)	143 ± 6	122 ± 5*
Stroke volume (ml/beat)	0.35 ± 0.01	0.31 ± 0.01*
FS (%)	49.3 ± 1.29	15.5 ± 0.89‡
dP/dt_max_ (mm Hg/s)	8819 ± 475	6970 ± 464*
dP/dt_min_ (mm Hg/s)	-8595 ± 495	-5888 ± 362‡
HR (beats/min)	415 ± 8	401 ± 9
BW (g)	440 ± 11	439 ± 19
HW (mg)	966 ± 21	1232 ± 34‡
HW/BW	2.21 ± 0.05	2.81 ± 0.07‡
LVW (mg)	741 ± 17	909 ± 16‡
RVW (mg)	160 ± 3	209 ± 17‡
AW (mg)	65 ± 3	114 ± 8‡
LW (mg)	939 ± 30	1143 ± 78†

LVESP: left ventricle end-systolic pressure; LVEDP: left ventricle end-diastolic pressure; LVESD: left ventricle end-systolic diameter, LVEDD: left ventricle end-diastolic diameter; E/A: ratio between LV E and A waves; Tau: LV relaxation constant; FS: fractional shortening; HR: heart rate; BW: body weight; HW: heart weight; LVW: left ventricle weight; RVW: right ventricle weight; AW: atrial weight; LW: lung weight.

* *P* < 0.05,

† *P* < 0.01,

‡ *P* < 0.001 *versus* sham (Wilcoxon test).

Up to now, only few studies have measured plasma cardiac markers in experimental animal models of HF [19, 20] and none in the same experimental model as we used. During the past few years, several candidate biomarkers have been studied in HF patients that can be divided into seven categories, markers of inflammation, oxidative stress, extracellular matrix remodelling, myocyte injury, myocyte stress, neurohormones, and renal dysfunction [21]. The four cardiac markers chosen were selected because they represent different pathways potentially implicated in the pathogenesis of LV remodelling: BNP is elevated in response to increased cardiac load; TnI elevation may indicate myocyte injury; CRP is a marker of inflammation and TIMP-1 is involved in matrix remodelling. Increased levels of each of these biomarkers are associated with major cardiac events in humans with only limited information on their association with LV remodelling [14, 22–24].

**Fig. 1:**
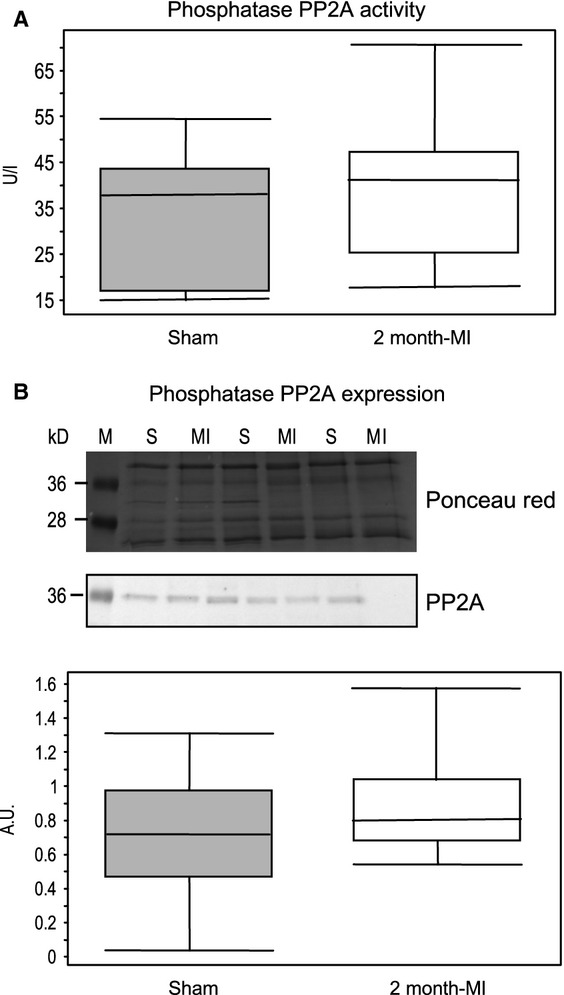
Quantification of phosphatase PP2A activity (**A**) and expression (**B**) in plasma of sham- and 2-month-myocardial infarction (MI) rats. (**A**) PP2A activity (U/L) was quantified in plasma (1 μl) by ELISA. (**B**) Representative western blot of PP2A expression in plasma samples (1 μl) of sham- (S) and 2-month-MI rats. Equal protein loading was verified by ponceau red staining of the membranes. The positions of size markers (M) are indicated on the left. Results for PP2A activity (**A**) and PP2A expression (**B**) in sham- (*n* = 16) (grey boxes) and 2-month-MI rats (*n* = 19) (white boxes) are expressed as box and whisker plots showing median (line) and quartile ranges.

**Fig. 2:**
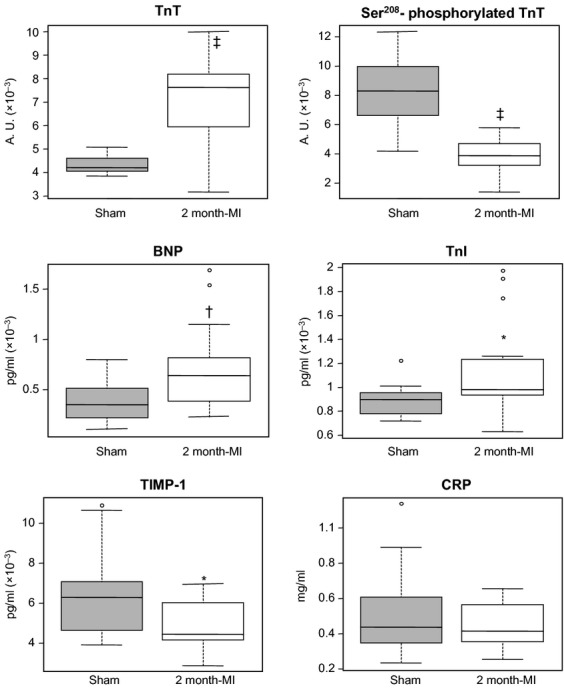
Cardiac biological protein parameters in sham- (*n* = 16) and 2-month-myocardial infarction (MI) rats (*n* = 19). TnT and P-Ser^208^-TnT were quantified by western blot as previously described [13]. B-type natriuretic peptide, troponin I, tissue inhibitor of metalloproteinase I and C-reactive protein were quantified using multiplex assays. Results for sham- (grey boxes) and 2-month-MI (white boxes) rats are expressed as box and whisker plots showing median (line) and quartile ranges; open circles indicate outliers. *P < 0.05 *versus* sham; †*P* < 0.001 *versus* sham; ‡*P* < 0.0001 *versus* sham.

We used the multiplex technological approach to quantify BNP, TnI, CRP and TIMP-1. This new approach has a higher dynamic range and the same sensitivity than a classical ELISA, with minimized sample volume requirements and respectively a sensitivity of 5 pg/ml for BNP, 5.7 pg/ml for CRP, and 158 pg/ml for TIMP-1. TnT and P- Ser^208^-TnT were quantified by western blot as previously described [13]. We verified by a calibration curve (range 0.25–2.5 μl) the limit of detection and linearity of the quantification by western blot for TnT (*R*^2^ = 0.805) and for P- Ser^208^-TnT (*R*^2^ = 0.93). The availability of pre-operative (1 day before surgery) plasma for both sham- and 2-month-MI rats allows the determination of normal plasma values for all these proteins, which is impossible in humans. For each protein, we did not see any difference in pre-operative plasma levels between the two groups of animals (data not shown). As we have previously reported, there was a significant increase in TnT level and a significant decrease in P-Ser^208^-TnT level in the plasma of 2-month-MI rats. We also quantified the activity and expression of phosphatase PP2A in plasma of sham- and 2-month-MI rats and we did not find any significant differences between the two groups (Fig. 1), excluding that an excess of phosphatase in the plasma of 2-month-MI rats [37.4 (24.8–50) in sham- *versus* 41.2 (26.2–53.3) U/l in HF-rats] could be involved in the decrease of phosphorylated form of TnT. For the other cardiac markers, we observed a significant increase in BNP [351 (325–377) in sham- *versus* 642 (600–684) pg/ml in HF-rats] and TnI levels [898 (881–915) in sham- *versus* 979 (940–1018) pg/ml in HF-rats] and a significant decrease in TIMP-1 level [6288 (6006–6570) in sham- *versus* 4430 (4295–4565) pg/ml in HF-rats] in plasma of 2 month-MI rats, but no modulation of CRP [898 (881–915) in sham- *versus* 979 (940–1018) μg/ml in HF-rats] (Fig. 2).

Plasma BNP was measured previously in different experimental models of hypertension. In renovascular hypertension, circulating BNP levels were found to be significantly increased [25]. Comparison of the normotensive strain, WKY and the hypertensive strain, SHR showed a threefold increase in circulating BNP levels in SHR rats, which were also enhanced by DOCA-salt treatment [26]. Using the same experimental model of DOCA-salt hypertension, plasma TIMP-1 levels were also found to be increased [19].

### Performance of P-Ser^208^-TnT compared to conventional cardiac markers

To determine which of the circulating biomarkers are able to predict LV remodelling, we compared the performance of each marker measured in our experimental 2-month-MI rat model by ROC curve analysis. P-Ser^208^-TnT and TnT were classified as excellent for accuracy, with an area under curve (AUC) of 0.951 and 0.921, respectively. Surprisingly, ROC curve analysis showed that BNP, TnI and TIMP-1 were less accurate with an AUC of 0.778, 0.750 and 0.697, respectively. Interestingly, CRP levels failed to discriminate between the two groups with an AUC of 0.474 (Table 2). In summary, P-Ser^208^-TnT with 94.7% sensitivity and 87.5% specificity was considered the best predictor of LV remodelling post-MI, suggesting the importance of P-Ser^208^-TnT as a new biomarker in HF (Table 2). These data are in accordance with previous publications showing that BNP and TIMP-1 plasma levels are also associated with severity of hypertension and not specifically to HF [19, 25, 26].

**Table 2:** Performance of phosphorylated Ser^208^-TnT as plasma biomarker predictive of cardiac remodelling

Biomarker	Cut-off	Sensitivity (%)	Specificity (%)	AUC
P-Ser^208^-TnT (A.U.)	5392	94.7	87.5	0.951
TnT (A.U)	5294	89.5	100	0.921
BNP (pg/ml)	534	72.2	81.2	0.778
TnI (pg/ml)	919	83.3	64.3	0.750
TIMP-1 (pg/ml)	6115	78.9	56.2	0.697
CRP (mg/ml)	3.78	68.4	37.5	0.474

AUC: area under curve; A.U.: arbitrary unit.

Next, for the most accurate biological markers of LV remodelling post-MI, P- Ser^208^-TnT and TnT, we investigated the relations between plasma levels and echocardiographic and haemodynamic parameters. We also performed the same analysis for BNP, which has been characterized in many studies in humans. We observed statistically significant relations between most of the functional parameters tested and P-Ser^208^-TnT, TnT and BNP (Table 3). Interestingly, only BNP (*P* = 0.046) and TIMP-1 (*P* = 0.014) levels were associated with stroke volume. In accordance with the calculated ROC curves, TnI was only found related to fractional shortening (*P* = 0.039) and as expected, none of the parameters were related to CRP levels. Current available information regarding correlation between these biological parameters and cardiac function only concerns BNP and TIMP-1. Data on BNP levels are contrasted depending on the experimental model used. Indeed, plasma BNP levels were correlated with dP/dt_max_ in rats with renovascular hypertension [25], while in nephrectomised rats, the increased circulating BNP levels were not associated with cardiac dysfunction [20]. Kramer *et al*. [19] found a correlation between TIMP-1 levels and dP/dt_max_ and heart weight-to-body weight, which was not found in our study – however this result was obtained in a DOCA-salt hypertensive model, which might in itself influence metalloproteinase pathways. In any case, our data clearly show the performance of P-Ser^208^-TnT and TnT to correlate with echocardiographic, haemodynamic and morphological changes linked to HF development.

**Table 3:** Relations of plasma phosphorylated Ser^208^-TnT, TnT and BNP to echographic and haemodynamic parameters

	P-Ser^208^-TnT TnT BNP								
	
Cardiac parameters	Rho	95% CI	*P* value	Rho	95% CI	*P* value	Rho	95% CI	*P* value
LVEDP (mm Hg)	−0.495	−0.662 to −0.213	0.003	0.743	0.436–0.834	4.94 10^−7^	0.402	0.034–0.610	0.020
LVEDD (mm Hg)	−0.633	−0.803 to −0.536	5.84 10^−5^	0.704	0.592–0.881	3.31 10^−6^	0.421	0.201–0.719	0.013
LVESD (mm)	−0.651	−0.838 to −0.565	3.09 10^−5^	0.699	0.604–0.891	4.28 10^−6^	0.396	0.195–0.676	0.020
Stroke volume (ml/beat)	0.261	0.064–0.589	0.137	−0.221	−0.537 to −0.063	0.209	−0.344	−0.427 to −0.044	0.046
Cardiac output (ml/min)	0.304	0.059–0.634	0.081	−0.174	−0.515 to 0.053	0.325	−0.219	−0.386 to 0.088	0.212
dP/dt_max_ (mm Hg/s)	0.288	−0.120 to 0.526	0.105	−0.513	−0.701 to −0.234	0.003	−0.168	−0.450 to 0.149	0.358
dP/dt_min_ (mm Hg/s)	−0.427	−0.587 to −0.054	0.016	0.639	0.356–0.699	0.0001	0.256	−0.123 to 0.412	0.165
FS (%)	0.657	0.569–0.868	2.41 10^−5^	−0.673	−0.874 to −0.579	1.30 10^−5^	−0.363	−0.624 to −0.194	0.035
E/A	−0.606	−0.664 to −0.023	0.0005	0.707	0.334–0.806	1.81 10^−5^	0.274	−0.232 to 0.548	0.150
Tau (ms)	−0.515	−0.640 to −0.285	0.003	0.671	0.241–0.744	2.62 10^−5^	0.358	−0.117 to 0.380	0.048

LVEDP: left ventricle end-diastolic pressure; LVEDD: LV end diastolic diameter; LVESD: LV end-systolic pressure; dP/dt_max_: cardiac contractility; dP/dt_min_: cardiac relaxation; FS: fractional shortening; E/A: ratio between LV E and A waves; Tau: LV relaxation constant; Rho: linear Spearman coefficient of correlation.

### Pharmacological modulation of heart failure

Finally, we assessed whether the biological markers that we characterized might be helpful in evaluating the beneficial effect of a known pharmacological treatment of HF, and we compared their sensitivity with that of echocardiographic and haemodynamic measurements. For this purpose, we chose to administer the If current inhibitor ivabradine, for which we previously demonstrated the beneficial effects in the same model of HF [11]. In fact, these preclinical data were recently confirmed in a large scale study of HF patients [27]. The beneficial effects of If inhibition are linked to its capacity to selectively reduce heart rate, without effects on cardiac contractility or conduction, leading to increased LV filling and stroke volume, improved diastolic coronary and improved coronary perfusion. Thus, we have been suggested that these pure beneficial haemodynamic effects would translate in a reduced expression of the tested biomarkers of HF severity.

For this purpose, a total of 14 rats were subjected to coronary ligation and received either vehicle or ivabradine (10 mg/kg/day) for 2 months, starting 1 week after ligation. This protocol was similar to that used in our previous study [15], except for a shorter duration of treatment (2 months *versus* 3 months), to correspond to the time course of our biomarker study. Table 4 details echocardiographic and haemodynamic parameters measured at 2 months, and shows that the treatment induced as expected a significant decrease in heart rate. This heart rate lowering effect was accompanied by significant increases in fractional shortening and stroke volume. The increased fractional shortening appeared related to the decrease (although non-significant) in LV end systolic diameter, in the absence of changes in LV end diastolic diameter. In parallel, ivabradine induced significant decreases in LV end diastolic pressure and the relaxation constant tau. Thus, despite the shorter treatment period, the present results confirm that ivabradine improves LV function in this rat MI model.

**Table 4:** Echographic, haemodynamic and histomorphometric parameters in 2-month-MI rats treated or not by an If current inhibitor ivabradine

Parameters	Untreated (*n* = 6)	Ivabradine (*n* = 8)
LVESP (mm Hg)	118 ± 10	117 ± 6
LVEDP (mm Hg)	8.8 ± 1.6	4.4 ± 0.9*
LVESD (mm)	9.17 ± 0.22	8.54 ± 0.16
LVEDD (mm)	10.35 ± 0.31	10.41 ± 0.17
Tau (ms)	9.56 ± 0.78	7.14 ± 1.50
Cardiac output (ml/min)	106 ± 4	110 ± 7
Stroke volume (ml/beat)	0.27 ± 0.01	0.33 ± 0.02*
FS (%)	11.3 ± 1.0	18.0 ± 1.1†
dP/dt_max_ (mm Hg)	7311 ± 553	7432 ± 609
dP/dt_min_ (mm Hg)	−5851 ± 532	−6280 ± 493
HR (beats/min)	393 ± 16	346 ± 10†
BW (g)	467 ± 19	445 ± 14
HW (mg)	1426 ± 87	1345 ± 37
HW/BW	3.05 ± 0.12	3.03 ± 0.09
LVW (mg)	949 ± 20	927 ± 20
RVW (mg)	295 ± 53	266 ± 17
AW (mg)	182 ± 29	152 ± 19
LW (mg)	1354 ± 118	1220 ± 101

LVESP: left ventricle end-systolic pressure; LVEDP: left ventricle end-diastolic pressure; LVESD: left ventricle end-systolic diameter; LVEDD: left ventricle end-diastolic diameter; E/A: ratio between LV E and A waves; Tau: LV relaxation constant; FS: fractional shortening; dP/dt_max_: cardiac contractility; dP/dt_min_: cardiac relaxation; BW: body weight; HW: heart weight; LVW: left ventricle weight; RVW: right ventricle weight; AW: atrial weight; LW: lung weight.

* *P* < 0.05,

† *P* < 0.01 *versus* untreated (Wilcoxon test).

**Fig. 3:**
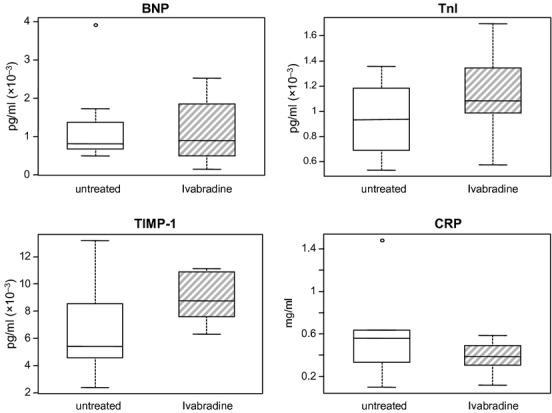
Cardiac biological protein parameters in 2-month-myocardial infarction (MI) rats untreated (*n* = 6) and treated by ivabradine (*n* = 8). C-reactive protein, B-type natriuretic peptide, tissue inhibitor of metalloproteinase I and troponin I were quantified by multiplex assays in plasma of 2-month-MI rats untreated (white boxes) and treated by ivabradine (hatched boxes). Results are expressed as box and whisker plots showing median (line) and quartile ranges; open circles indicate outliers #*P* < 0.05 *versus* 2-months-MI.

**Fig. 4:**
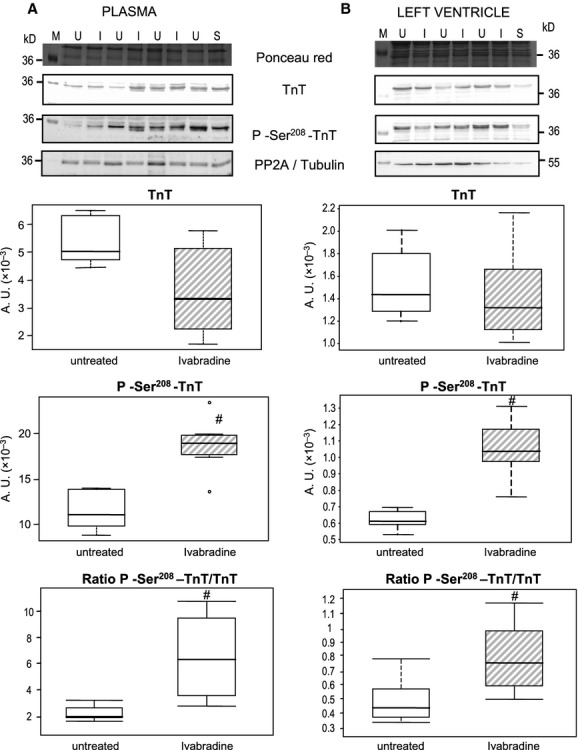
Effect of Ivabradine treatment on plasma and left ventricular (LV) TnT and P- Ser^208^-TnT levels of rats 2 months after myocardial infarction (MI). Representative western blot of plasma (**A**) and LV (**B**) samples. Equal protein loading was verified by ponceau red staining of the membranes and PP2A staining for plasma proteins and tubulin for LV proteins. TnT and P-Ser^208^-TnT were quantified in the plasma (1 μl) and LV (50 μg) of MI (*n* = 6) (white boxes) and MI treated by Ivabradine (*n* = 8) (hatched boxes) rats at 2 months. The positions of size markers (M) are indicated on the left for plasma samples and on the right for LV samples. U: untreated; I: ivabradine treated; S: standard used for normalization. Results for TnT and P-Ser^208^-TnT are expressed as box and whisker plots showing median (line) and quartile ranges; open circles indicate outliers, except for the ratio of P-Ser^208^-TnT to TnT. #*P* < 0.05 *versus* 2-months-MI.

Then we investigated the influence of ivabradine treatment on the plasma levels of cardiac biomarkers that we have previously tested in our experimental model of MI. BNP, TnI, TIMP-1 and CRP were not affected by the treatment (Fig. 3). B-type natriuretic peptide data are in accordance with a previous publication [28]. Interestingly, we observed a significant increase of plasma P-Ser^208^-TnT levels with no significant changes in TnT levels in 2-month-MI rats treated by ivabradine compared to the 2-month-MI untreated rats (Fig. 4A), suggesting the potentiality of P- Ser^208^-TnT as biomarker of a pharmacological improvement of cardiac function in HF. We also compared the levels of TnT and P-Ser^208^-TnT in the LV of the same animals to determine whether the plasma circulating levels are a reflect of cardiac changes. We did not observe any significant modification of total TnT in LV of treated *versus* untreated 2-month-MI rats (Fig. 4B). In contrast, ivabradine induced a significant increase in LV P-Ser^208^-TnT correlated to the circulating plasma levels (Fig. 4B). Interestingly, a significant correlation (*P* = 0.026) was observed between plasma and VG P-Ser^208^-TnT levels. These data suggest that the phosphorylation level of TnT, quantified in plasma by the ratio P-Ser^208^-TnT to TnTis an indicator of the changes occurring at the level of the heart.

## Conclusions

In our experimental model, we found modulation by HF of BNP, TnI and TIMP-1 plasma levels as shown in humans [10]. Despite their modulation, they were less sensitive and specific than P-Ser^208^-TnT for the presence of HF. Interestingly, P-Ser^208^-TnT was shown to be associated with most of the echographic and haemodynamic parameters of cardiac function. Our data also suggested that P-Ser^208^-TnT may be a biomarker of a pharmacological improvement of cardiac function in HF. For the future studies, development of a standardized assay for quantification of TnT and P-Ser^208^-TnT will be helpful. Taken together, these results suggest that P-Ser^208^-TnT is a sensitive biomarker of HF in this preclinical model.

## Fundings

This study was supported by grants from Inserm-transfert (2010), “Appel d'offres Recherche translationnelle “DHOS-Inserm (2010) and the Agence Nationale de la Recherche 2010 (ANR blanc-SVSE1).
